# System Dynamics Modeling in the Evaluation of Delays of Care in ST-Segment Elevation Myocardial Infarction Patients within a Tiered Health System

**DOI:** 10.1371/journal.pone.0103577

**Published:** 2014-07-31

**Authors:** Luciano de Andrade, Catherine Lynch, Elias Carvalho, Clarissa Garcia Rodrigues, João Ricardo Nickenig Vissoci, Guttenberg Ferreira Passos, Ricardo Pietrobon, Oscar Kenji Nihei, Maria Dalva de Barros Carvalho

**Affiliations:** 1 Department of Health Sciences, State University of Maringa, Maringa, Parana, Brazil; 2 Division of Emergency Medicine, Department of Surgery, Duke Global Health Institute, Duke University, Durham, North Carolina, United States of America; 3 Nucleus of Data Processing, State University of Maringa, Maringa, Parana, Brazil; 4 Graduate Program in Informatics - PPGIA, Knowledge Discovery and Machine Learning Group, Pontificia Universidade Católica - PUC-PR, Curitiba, Parana, Brazil; 5 Department of Nursing, State University of the West of Parana, Foz do Iguaçu, Parana, Brazil; 6 Instituto de Cardiologia do Rio Grande do Sul - Fundação Universitária de Cardiologia, Porto Alegre, Rio Grande do Sul, Brazil; 7 Department of Medicine, Faculdade Ingá, Maringa, Parana, Brazil; 8 Department of Informatics, Brasilia University, Brasilia, Brazil; 9 Department of Surgery, Duke University Health System, Durham, North Carolina, United States of America; University of Bologna, Italy

## Abstract

**Background:**

Mortality rates amongst ST segment elevation myocardial infarction (STEMI) patients remain high, especially in developing countries. The aim of this study was to evaluate the factors related with delays in the treatment of STEMI patients to support a strategic plan toward structural and personnel modifications in a primary hospital aligning its process with international guidelines.

**Methods and Findings:**

The study was conducted in a primary hospital localized in Foz do Iguaçu, Brazil. We utilized a qualitative and quantitative integrated analysis including on-site observations, interviews, medical records analysis, Qualitative Comparative Analysis (QCA) and System Dynamics Modeling (SD). Main cause of delays were categorized into three themes: a) professional, b) equipment and c) transportation logistics. QCA analysis confirmed four main stages of delay to STEMI patient’s care in relation to the ‘Door-in-Door-out’ time at the primary hospital. These stages and their average delays in minutes were: a) First Medical Contact (From Door-In to the first contact with the nurse and/or physician): 7 minutes; b) Electrocardiogram acquisition and review by a physician: 28 minutes; c) ECG transmission and Percutaneous Coronary Intervention Center team feedback time: 76 minutes; and d) Patient’s Transfer Waiting Time: 78 minutes. SD baseline model confirmed the system’s behavior with all occurring delays and the need of improvements. Moreover, after model validation and sensitivity analysis, results suggested that an overall improvement of 40% to 50% in each of these identified stages would reduce the delay.

**Conclusions:**

This evaluation suggests that investment in health personnel training, diminution of bureaucracy, and management of guidelines might lead to important improvements decreasing the delay of STEMI patients’ care. In addition, this work provides evidence that SD modeling may highlight areas where health system managers can implement and evaluate the necessary changes in order to improve the process of care.

## Introduction

In 2011, approximately 7 million people died worldwide from ischaemic heart disease, representing 11.2% of all global deaths. While this issue primarily affects developed countries it is becoming increasingly problematic in developing countries [1]–[2]. ST-segment elevation myocardial infarction (STEMI) is the most lethal form of acute coronary syndrome (ACS). In addition to high mortality rates, STEMI is also associated with high rates of serious complications that can be avoided with early treatment. Primary percutaneous coronary intervention (PCI), recognized worldwide as the best strategy for myocardial reperfusion in patients with STEMI, improves patients’ survival and quality of life. However, expediting PCI continues to be a constant challenge for cardiovascular specialists and health systems worldwide [3]–[6].

The American College of Cardiology/American Heart Association and European Society of Cardiology recommend a goal time from first medical contact (FMC) to balloon catheter inflation at a PCI center (FMC-to-device) of less than 120 minutes for patients who call requesting emergency medical services or self-present to a non-PCI-capable hospital [2], [7]. There is a positive correlation between the FMC-to-device time and mortality rates of STEMI patients that is attributed to delays in care, which limit the benefits of PCI [6]–[9]. Studies show that multiple factors are responsible for these delays including the regional and patient characteristics, like geographic, demographic, behavioral, transportation availability, and the structure of the referral services [10]–[15].

Even in developed countries, most patients with STEMI present to a primary hospital without PCI capabilities and it is the rapid transport from a primary hospital to a tertiary hospital with PCI that is a major obstacle [16]–[18]. The College of Cardiology/American Heart Association and European Society of Cardiology recommend a goal time to proper care of STEMI patients from Door In at a primary hospital to the departure to a PCI Center (Door-in-Door-out time) of 30 minutes or less [2], [7]. The ideal time from patient entrance at the primary hospital (Door In) until the acquisition of 12-lead ECG reviewed by physician is 10 minutes, and an additional 20 minutes are recommended for the ECG transmission, decision to transfer a STEMI patient based on PCI center feedback and the patient departure (Door Out). A systematic review revealed that only 25% of United States hospitals have catheterization capacity and transportation of STEMI patients to a catheterization capable hospital is often not within the recommended 30 minutes timeframe. This is often due to geographic variations and diagnosis delays [19]. Other studies acknowledge the delays with STEMI patients from primary hospitals, but do not speculate which variables at the primary hospital are responsible for delays [20]–[21].

In Brazil, the proportion of STEMI patients diagnosed by prehospital and hospital triage systems is unclear. There are a few studies in Brazil reporting the demographics of those who received cardiac reperfusion therapies. In a 2009 survey of 158 STEMI patients from Rio de Janeiro, 67.7% arrived at the hospital within 180 minutes, 81.3% within 360 minutes and 8.4% arrived after twelve hours. In this study, 26% of patients were treated with PCI, 32% with thrombolytics, and 42% with optimal medical therapy. Importantly, about 35% of STEMI patients who should have received thrombolysis did not receive it in this study [22].

Health system evaluations commonly gather some of the following information: screening time, time to completion of ECG and time to transfer to an interventional cardiology center [23]–[24]. However, to our knowledge no other study has analyzed these variables through System Dynamics Modeling [25] and sensitivity analysis [26] to provide a more complete in-depth health system evaluation. As such, the intent of this study is to evaluate delays in routine emergency care of STEMI patients using on-site observations, QCA, system dynamics modeling and sensitivity analysis in order to perform an in depth health system evaluation. This evaluation will enable a better understanding of the variables related to treatment delays in STEMI patients and identify the necessary improvements in order to provide adequate care according to the recommended guidelines.

## Methods

### Hospital Setting

This research was conducted at João Samek primary hospital located in Foz do Iguaçu, Parana, Brazil. This hospital has an Emergency Care Unit, open 24 hours per day 7 days per week with the capacity to treat 300 to 400 patients per day [27]. Patients diagnosed with STEMI at this hospital are transported by emergency medical services (EMS) to Minister Costa Cavalcanti tertiary hospital and regional interventional cardiology referral center (PCI center), located in Foz do Iguaçu. This regional PCI center attends approximately 170 patients per month, referred from different health care institutions, and on average performs 90 primary percutaneous coronary interventions per year. The distance between the primary hospital and the PCI center is 2.05 miles.

### Study Period

The study was conducted from August 2011 to October 2012.

### Institutional Review Board

All participants provided a written informed consent to participate in this study. This study was approved by the Institutional Review Board at the State University of Maringa (COPEP/UEM), in Brazil, under the registration number 266/2011.

### Qualitative Data Analysis

The qualitative research was conducted and reported based on the ‘consolidated criteria for reporting qualitative research’ (COREQ) [28].

#### Data Source

Our qualitative approach included interviews with health professionals regarding their perception of the care of patients admitted with chest pain and diagnosed with a STEMI who required transfer to a PCI center. The study subjects were health professionals who work at the João Samek primary hospital in Foz do Iguaçu. A convenience sampling was used in the selection of participants. The principal investigator (LA) interviewed two physicians, two nurses and two managers. These health professionals were enrolled to participate in this study during the period of September and October 2012. The nurses and physicians had experience providing care in triage and the emergency department while the managers were administrators for the primary hospital. In order to be included in the study, the health professionals had to have worked at the primary hospital for at least two years and have interest in participating in the research project. All participants had more than 10 years of experience in healthcare and 2 with more than 30 years of experience.

Study participants did not know the principal investigator beforehand, and vice versa. The script for the interviews included two open ended questions: *1) In your opinion, what are the causes of delay in care and transfer of patients with STEMI from a primary hospital to the PCI center?; 2) What measures do you think could reduce attendance time and transfer of a patient with STEMI to the PCI center?*

#### Interview Procedures

All interviews were performed individually and occurred in a private room within the primary hospital facilities. All interviews were recorded and on average lasted 20 minutes. The speakers were identified by E1, E2 (nurses) and M1, M2 (physicians), and G1, G2 (Managers). Data collection occurred during the months of September and October 2012.

#### Data Transcription

In order to improve accuracy, all transcriptions were performed by the principal investigator no more than seven days from the original interview. Data saturation was achieved at the conclusion of 6 interviews. All interviews were transcribed verbatim. Notes and impressions obtained during participant interviews were added to the original transcription as comments. All transcripts were reviewed for accuracy and were available to other participating researchers. Physician, nurses and managers identifiers were removed from the transcript to ensure confidentiality. Quotations used for illustration in this report were translated from Portuguese to English and independently back-translated by other bilingual researchers to ensure translation accuracy. Transcripts of the interviews were sent to participants for comments and changes for quality control.

#### Data Analysis

Interview analysis was completed utilizing content analysis, a research technique used to objectively categorize communication content allowing researchers to identify the essential information behind each participant narrative [29]–[32]. There are three main phases of content analysis: pre-analysis, material exploration and inference/interpretation of results [30], [32]. This study included: a detailed reading of the interview transcript (pre-analysis); data categorization and inclusion of descriptive quotations (material exploration); and interpretation of results (inference/interpretation).

Transcriptions were coded using a combination of manual coding and NVIVO software, version 10.0 (QSR International, Melbourne, Australia) [33]. The data were organized in a spreadsheet Excel (Microsoft Office, Microsoft Corporation, USA) and exported to.csv files (comma-separated values). Data were analyzed and the absolute and relative frequencies of the categories that emerged from the interviews of health professionals were presented in Polar Axis graphs (R software, version 2.15.0) [34].

### Qualitative Comparative Analysis (QCA)

QCA was performed for a formal analysis of qualitative evidence using Boolean Algebra rather than correlational methods [35]. QCA is a configurational comparative technique that is half-way between the qualitative and quantitative approach [35]–[36]. QCA has the advantage of being able to analyze samples in small-N situations forming sets of variables based on half verbal/conceptual and half logical/mathematical criteria. The main purpose of QCA is to detect different configurations of the variables that lead to an outcome of interest, allowing a better understanding of a complex multivariable scenario. Thus the QCA focus on the analysis of multivariable conditions necessary and/or sufficient for an outcome of interest unraveling equifinality, multifinality or asymmetric structural causalities [36]–[39]. Thus, there are three analytic components of QCA: necessity analysis; sufficiency analysis; data set calibration [35], [40]–[41].

For this study, we used crisp-set QCA (csQCA), a conventional and most commonly used technique of QCA, which generates explanatory models containing one or more causal paths to the possible outcomes of interest based on the evidence (variables) [40]–[42].

In our study, the data for QCA were obtained from medical records of patients initially admitted to the emergency department at the primary hospital with a confirmed STEMI diagnosis who were then transferred to the PCI center in Foz do Iguaçu between August 1^st^, 2011 and October 30^th^, 2012. We identified and included the medical records of 29 STEMI patients. The main data obtained from the STEMI patients medical records were four variables: First Medical Contact [FMC] that was defined as the first contact of the STEMI patient with the nurse (in most of the cases) and/or physician (in emergency cases) at the primary hospital; Electrocardiogram acquisition [ECG]; ECG transmission and PCI center team feedback time [TXF]; Patient’s Transfer Waiting Time [TWT]; and two outcomes (Ejection Fraction <50% [EF]; Length of Stay [LOS]).

We constructed a matrix with binary variables, with ‘zero’ corresponding to ‘no delay’ and ‘one’ corresponding to a ‘delay in the initial care of patients with STEMI’. We then transformed this array into a truth table, a critical part of a QCA analysis, which evaluates for causal combinations that are sufficient for the outcome and are represented in letters that are linked with Boolean operators [35]–[38]. The three basic Boolean operators are logical OR (+), logical AND (*), and logical NOT (where the negative is customarily denoted in QCA by replacing an uppercase letter with a lowercase letter). So in the next step, the Boolean minimization was performed which excludes some of these causal combinations, allowing the identifications of the so called ‘prime implicants’ (PI) related with the regularities in the data [41]. These PI state what combinations of conditions are necessary or sufficient for the outcome to occur or not [35], [39], [43].

A function within the package QCA in the R software that performs this boolean minimization is the “eqmcc”, from which one can derive different solutions from the truth table of the object or a suitable dataset: 1) Complex solutions (Detailed sets); 2) Parsimonious solutions (incorporate logical remainders into the minimization process, generating only the essential simplified sets); 3) Intermediate solutions (middle way path between Complex and Parsimonious solutions) [40]–[42].

### Primary Hospital Process Measures

Different studies have proposed the analysis of additional time intervals within the Door-in Door-out time in order to better understand the processes related with the delay of STEMI patient care within the primary hospital [44]–[45]. In this work, we proposed to further divide the main two stages (Door-in to ECG acquisition; ECG transmission to patient departure) in four time intervals, considering an optimal time for each process, as follows: 1) Door-to-FMC: 2 minutes; 2) FMC-to-ECG: 8 minutes; 3) ECG transmission-to-PCI center team feedback: 5 minutes; 4) Patient’s transfer waiting time until departure to PCI center: 15 minutes. Since these four time intervals totalize the recommended 30 minutes, these intervals were utilized as the ideal times to obtain the delays in each stage.

### System Dynamics Modeling

System Dynamics (SD) is an approach to frame, describe and analyze complex systems allowing a better understanding of how their key constitutional elements interact to generate a specific outcome what may help to provide solutions to a problem [25], [46]–[47]. In SD, we create models using diagrams of stocks and flows, whose characteristic is to allow the model to be analyzed quantitatively. Stocks represent the state of the model entities at any point in time, and flows representing the rate of change of stock [25]. A dynamic complex system is characterized mainly by: 1) Interactions between the system’s constituents; 2) Dependency on time; 3) An internal complex causal structure subjected to feedbacks, and 4) Delayed behavioral reactions, which are difficult to predict [25]. In addition, the models using SD generally work with continuous time and change of levels of stocks based on instantaneous values.

According to the nature of this study, we build a model based on the concept of “Aging Chains” [48], a structure model that represents the different stages of a maturing process where attributes change as time goes. This structure has information about how long each modeled entity is retained at each stage and based on this a model of the impact on the final outcome of STEMI patients was created.

#### Model Creation and Mediated Modeling

The model was created to adjust the data based on interviews with the primary hospital professionals and from STEMI patients’ medical records. All modeling was conducted using Vensim DSS version 5.11 of Ventana Systems, Inc [49]. During model creation, mediated modeling methods were adopted to incorporate researchers and professionals knowledge and experience to produce a coherent, simple but elegant simulation baseline model and create strategies for the solution of a specific problem [50]. Through codification, and converting ideas from qualitative interviews into a SD model these data may indicate a strategic plan to decision makers.

#### Sensitivity Analysis and Model Calibration

We conducted a sensitivity analysis and a model calibration according to Oliva [51], which relies on Sterman [47]. The sensitivity analysis allow us to identify variation in the behavior of the model when changes are made to certain parameters. In this case the simulation is executed many times with different parameters until the increase of resources does not change the outcome of the model. The model calibration is an interactive process where the modeler, with the knowledge gained from the analysis of sensitivity, adjusts the parameters and re-executed the model and go back to the first step until the behavior of the model reach the best cost-benefits.

## Results

### Qualitative Data Coding of Delay Causes

A total of six health professionals from a primary hospital were interviewed. The qualitative data were subjected to content analysis and we identified the categories of causes of delay in the care and transfer of STEMI patients from the primary hospital to the PCI center in Foz do Iguaçu, Brazil.

**Figure 1:**
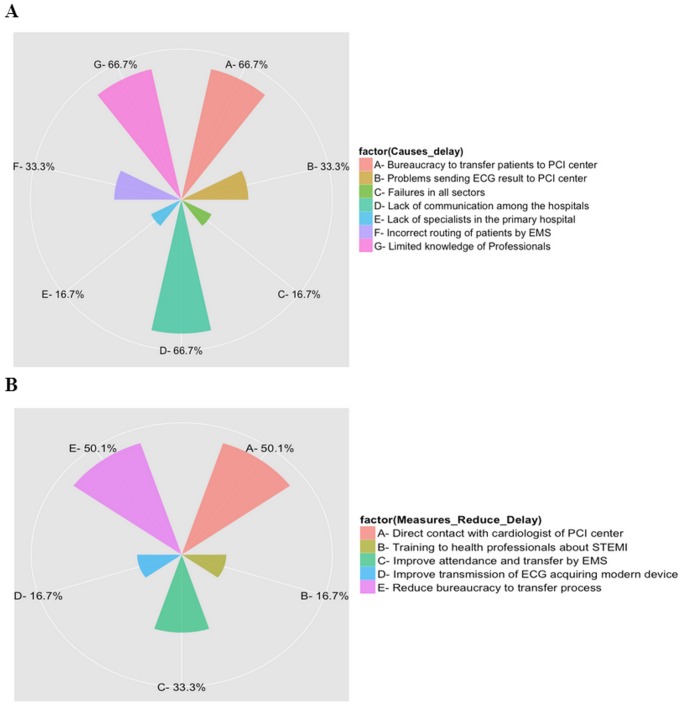
Qualitative data categories obtained from the interviews with primary hospital professionals: A) Causes of Delay; B) Measures to Reduce Delay.

When asked about the causes of delay in the care and transfer of STEMI patients from the primary hospital to the PCI center, the qualitative categories of responses were: a) professional; b) equipment and c) transportation logistics (Figure 1). Professional causes of delay included: professionals’ limited capacity to recognize STEMI patients (66.7%), lack communication between the primary hospital medical staff and the staff of the PCI center (66.7%) and lack of specialists in the primary hospital (16.7%). Transportation logistics category of causes of delays include: bureaucracy involved in transferring STEMI patients to the PCI center (66.7%), and incorrect routing of patients by EMS to the primary hospital instead of going straight to the PCI center (33.3%). Equipment causes of delays included: equipment problems sending the ECG result to the PCI center (33.3%). Finally, one participant reported ‘failures in all sectors’ (16.7%) (Figure 1A).

When the professionals were asked about possible measures to reduce transfer time of the STEMI patient from the primary hospital to the PCI center, the qualitative categories were: a) Professional Development (improve health professionals training and capacity to manage STEMI patients [16.7%], improve communication between the primary hospital and PCI center staff, mainly direct contact with cardiologists [50.1%]); b) Transportation logistics (reduce bureaucracy to transfer process [50.1%] as well as ensuring appropriate EMS disposition of STEMI patients to an PCI center [33.3%]); c) Equipment (acquiring appropriate equipment to improve communication [implementation of electrocardiography connected to internet] [16.7%]) (Figure 1B).

Below are excerpts of key interviews that played a role in defining the categories of causes of delay with reference to the question: *1) In your opinion, what are the causes of delay in care and transfer of patients with a STEMI from a primary hospital to the PCI center?*

*“We’ve discussed this a long time, there are flaws in all sectors; we realize that some employees are not prepared to act, difficulties in the initial recognition of patients with myocardial ischemia cause the delay from patient arrival until exit to another hospital” (E1).*

*“In patients with a diagnosis of STEMI, the physician attendance here is fast, then we fax the ECG, by phone warn the referral hospital that the ECG is being faxed, awaiting their response there is always a delay. When we call for feedback, they said that the fax is unreadable, it has artifact, ask if the ECG cables are placed correctly, and say that we have to repeat another ECG and send it again”(E2).*

*“The delay occurs for two reasons, one is that most of the physicians who work here are not qualified as emergency physicians and don’t have the required technical knowledge. They are here to get experience. The second reason is difficulty in patient transportation, and the bureaucracy in this process. Sometimes it would be appropriate [for EMS] to directly transport the patient to the Interventional Cardiology Center and it doesn’t occur.”(G1).*

Below are excerpts of key interviews that played a role in defining the categories of measures with reference to the question: *2) What measures do you think could reduce attendance time and transfer of a patient with STEMI to the PCI center?*

*“We need an educational refresher course performed by cardiologists from the PCI Center on ECG interpretation. We would be more secure and comfortable transferring patients [with this knowledge…]” (M2).*

*“The goal is to reduce the bureaucracy and obtain a modern ECG device. In my opinion the adoption of fax to transmit the ECG information increased the delay and decrease the quality of the information that is transmitted, which has hampered the patients’ transfer. Many times the complaint is that the ECG is unreadable” (G1).*

*“When there is an elevation in one or two leads of the ECG, but it is not certain, we talk with the emergency physician of the referral hospital, that also most often is not a cardiologist. He does not trust our word, asks to repeat the ECG, that was faxed which increases the delay. If the contact is direct with the interventionist cardiologist this would decrease the delay time in transfer”(M2).*

### Qualitative Comparative Analysis (QCA)

Our sample consisted of 29 patients transferred from the primary hospital to the PCI center for a STEMI. The sample was mostly made up of men (82.8%) with a mean age of 60±11 years and a body mass index of 27.8±5.9, mostly with incomplete primary education (58.6%), 41.4% had a family history of coronary artery disease, while 10.3% had a prior AMI. Sixty-nine percent had high blood pressure (hypertension), 37.9% had diabetes mellitus, 31.0% dyslipidemia, 41.4% were smokers and only 6.9% practiced physical activity. Most of FMC occurred between 6 pm until 12 am period (41.4%), followed by the afternoon period (24.1%).

To perform QCA we adopted the intermediate solution algorithm, and four variables (FMC, Electrocardiogram [ECG], ECG transmission and PCI center team feedback time [TXF], Transfer Waiting Time [TWT]) and two outcomes (Ejection Fraction <50% [EF]; Length of Stay [LOS]) were considered. These two outcomes were selected since higher LOS correlate with increasing the hospital expenses and EF loss is related to the infarct type (whether affecting the left [anterior descending artery and circumflex artery] and/or right coronary) and degree of left ventricular damage indicating mainly moderate and major infarcts.

The QCA showed that only one variable and one combination (set) generated by the intermediate solution algorithm, lead to the delay of STEMI initial treatment indirectly increasing the chance of reducing the first outcome analyzed, the ejection fraction (EF) less than 50%. In this case, the QCA generated the following expression: ecg*txf*TWT+FMC*ecg*TWT, which indicated that the reduction of EF are associated with delay in the following time periods: 1) TWT; or 2) FMC and TWT.

In addition, the QCA intermediate solution algorithm showed that two combinations of variables lead to an increase of the length of stay (LOS). In this case, the QCA generated the following expression: FMC*ecg*TXF*TWT+FMC*ECG*txf*TWT. The expression indicated that the increase of LOS may happened when there is a delay in the following combinations of time periods: 1) FMC, TXF and TWT; **or** 2) FMC, ECG and TWT. When evaluating increased LOS as an outcome, it was associated with a delay in any stages of care at the primary hospital. These data indicated that both LOS and EF are sensitive to TWT and FMC and in the case of LOS it is also affected by ECG and TXF.

### System Dynamic Modeling

**Figure 2:**
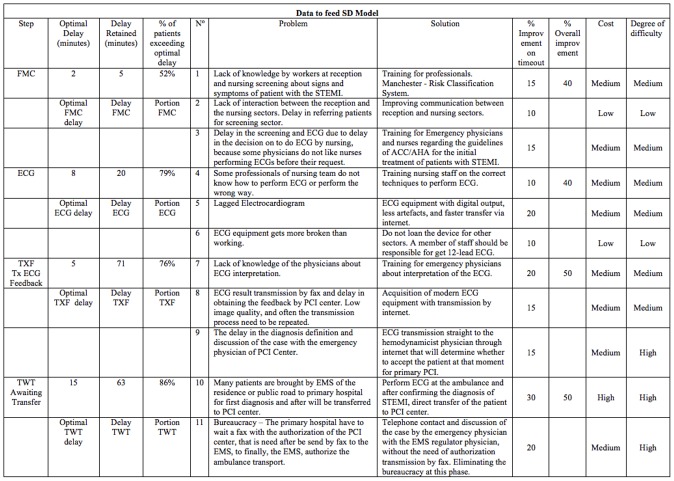
Data obtained by medical records and mediated modeling to feed SD model.

To start building the system dynamic model, the following time intervals, already described previously, were utilized: FMC, ECG, TXF and TWT. From the collected data a spreadsheet was created (Figure 2) with data from each step. Optimal timing, according to the literature, was inserted and the average delay, the delay retained in each step and the percentage of patients that exceeds the optimal delay was calculated. During the mediated modeling, we described the challenges at each step, the possible solutions and calculated the percentage of potential improvements (Figure 2). According to these data, the 3rd (TXF) and 4th steps (TWT) represented the variables that could, if improved, enable the highest percentage (50%) of overall improvement in order to decrease the delay of treatment of STEMI patients. Accordingly, the results also indicated that the 3rd and 4rd steps presented high percentage of patient exceeding delay, 76% and 86%, respectively, compared to the ideal time (Figure 2).

**Figure 3:**
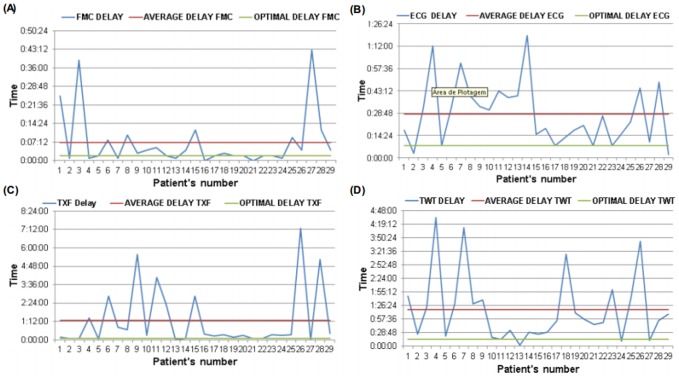
Representation of the Ideal Delay (green line), Average Delay (red line) and Patients Delay (blue line) in four different steps: (A) First Medical Contact (FMC), (B) ECG and diagnostic (ECG), (C) ECG transmission by fax and feedback from PCI center (TXF), (D) Transfer waiting time (TWT). Y axis represent the delay time (hour:minutes:seconds) and the X axis the number of each patient.

Based on collected data, we show in Figure 3 the optimal delay (green line), the average delay (red line) and the delay for each patient (blue line), in four different steps: FMC (3A), ECG diagnosis (3B), TXF (3C) and TWT (3D). The X axis present the number of each patient (1 to 29) and the Y axis represents the delay time in minutes. The 3rd step (TXF) presented 76% of the patients exceeding the optimal delay; the 4rd step (TWT) presented 86% and the 2nd step (ECG diagnosis) presented 79%.

**Figure 4:**
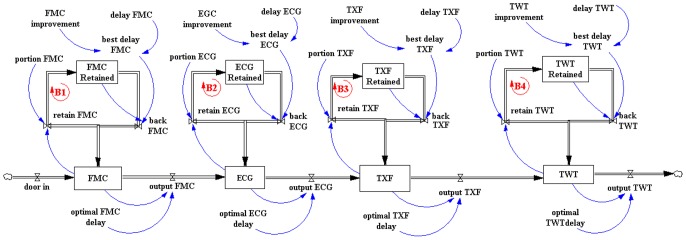
Systems Dynamics Model representing the routine to assist patients inside primary hospital considering the four different steps: First Medical Contact (FMC), ECG and diagnostic (ECG), ECG transmission by fax and feedback from PCI center (TXF) and Transfer waiting time (TWT).

We created a baseline simulation model (Figure 4) showing all the steps from the arrival of the patient until the transfer to a PCI center. In this model, the stocks represent the four stages that the STEMI patients need to pass within the primary hospital to be adequately treated namely “FMC”, “ECG”, “TXF” and “TWT” and flows represents the patients moving through each department while receiving care in the primary hospital. In the proposed model the STEMI patients are retained in each stage for a variable ‘x’ time, increasing their delay time in each stage and consequently increasing the Door in-Door out treatment time. In each stage, the percentage of necessary improvement is calculated in order to obtain a condition where the delay do not occur, what is considered the main goal, in order to reduce the retention of the STEMI patients in each stage. After mediated modeling, we created a diagram over these stocks that represents the actions to implement proposed improvements and reduce the time spent in each phase of patient care. These diagrams have a feedback loop (represented by B1, B2, B3 and B4), which means that if a variable is increased above a certain level it is automatically adjusted and as a result it will return to a desired level and vice versa.

### Model Validation and Policy Testing

**Figure 5:**
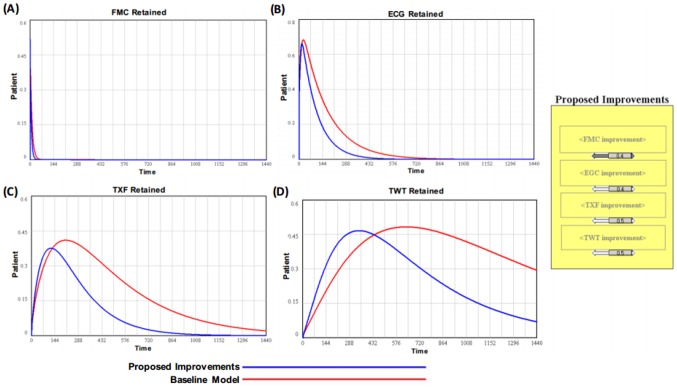
Dashboard to calibrate the model: X axis represents time patients are retained in each process and Y axis represents the percent of patients retained. The right box present the proposed improvement of each variable.

Based on analysis of collected data, model simulation and calibration it was possible to generate an interval of values that results in no delay (Table 1 and Figure 5). On this table, the data in the column “Value Estimate” is derived from the column “% overall Improvement” presented in the Figure 2, while the column “Interval of Uncertainty” is the variation of improvements obtained during certain number of simulations. This interval is necessary because in the real world there is always the possibility of unexpected events.

**Table 1:** System Dynamic Model calibration parameters for each step within the primary hospital.

Calibration SD Model - STEMI			
Step	Parameters	Value Estimate	Interval “appropriate”Degree of uncertainty
First Medical Contact (FMC)	FMC improvement	40%	10–50%
ECG and diagnostic (ECG)	ECG improvement	40%	10–50%
ECG Transmission by fax and feedback from PCI center (TXF)	TXF improvement	50%	10–60%
Transfer waiting time (TWF)	TWF improvement	50%	10–60%

In order to adjust the system according to the international guidelines, we tested some policies using different scenarios and values by manipulating variables and model parameters (Figure 5). The objective was to achieve a reasonable match between the observed and the simulated values and detect unacceptable errors, demonstrating expected improvement reducing the delay in care of STEMI patients. The red line represents the number of patients retained in each phase of baseline model and the blue line represents the same values after simulation of the proposed improvements (illustrated in Figure 2).

**Figure 6:**
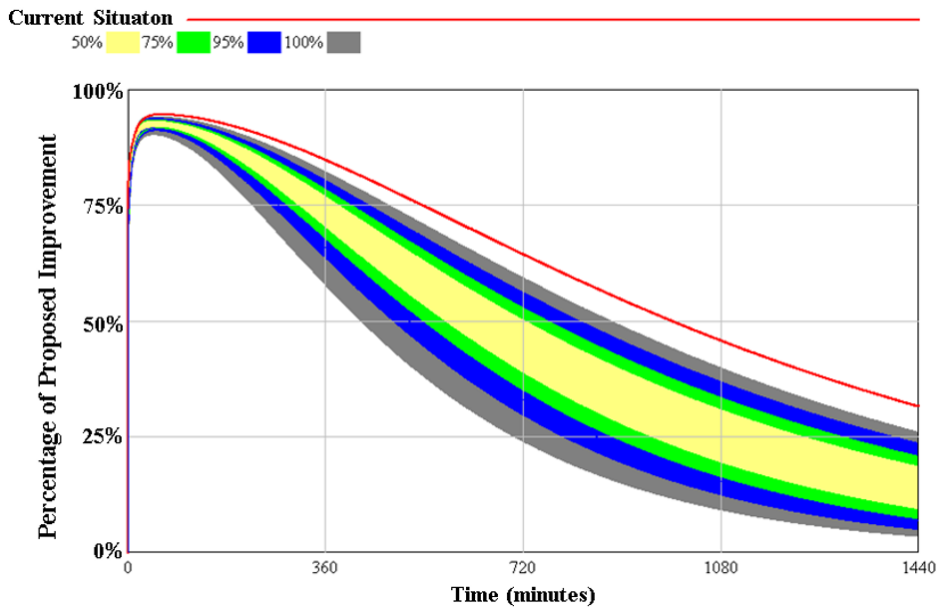
Sensitivity analysis for 20,000 simulations. Total retained means percent of patients (0 to 100%) retained on all processes.

We executed 20,000 simulations where the model parameters changed each time with the aim to understand the behavioral boundaries of a model and test the robustness of policies. The Figure 6, representing the proposed improvements, shows the reduction in the retention time of STEMI patients, by performing these simulations. It is expressed as the probability of occurrence, the range in yellow color indicates that 50% of the results obtained are in this range, 75% of the results are shown in the range yellow plus green, 95% are in the range green plus blue plus yellow band and 100% within the range of the results of all the tracks.

## Discussion

This is the first study to analyze the delays associated with care of STEMI patients and the factors behind such delays, using on-site observation, interviews, medical records analysis, qualitative comparative analysis (QCA), system dynamic modeling, mediated modeling and sensitivity analysis. This study identified four primary areas causing delays in treatment of STEMI patients from initial medical attention until their transfer from the primary hospital to the PCI center. These stages and their average delays in minutes were calculated based on the differences considering the gold standards recommended by the American Heart Association and European Cardiology Society [2], [7]. According to these recommendations, the ideal time for each of these stages were 10 minutes for FMC and ECG and 20 minutes for TXF and TWT, resulting in the Door-in Door-out time of less than 30 minutes. Based on these criteria, the average delays in each stage at the primary hospital investigated were: a) FMC: 7 min, b) ECG: 28 min, c) TXF: 75 min, d) TWT: 78 min. Through the interviews, we were also able to identify three factors related with these delays: a) professionals, b) equipment and c) the logistics of transporting the patient. Corroborating these findings, other studies also have concluded that delays in the treatment of STEMI patients are caused by many hospitals not having the proper conditions including primary resources of personnel, equipment and good logistical planning to achieve the Door-in Door-out according to the recommended guideline’s time of less than 30 minutes [2], [6], [52].

The qualifications of health professionals and the promotion of health education to the general population depends on the social, technological and economic development of each country [53]. In Brazil, different factors may be related to the unpreparedness of health professionals and lack of medical specialists in the public sector. These include: a) Inconsistent training of health professionals and the existence of gaps between the recommended national curriculum guidelines and the minimum curriculum applied by some universities; b) Disparities in the distribution of medical specialists in Brazil (between rural and urban areas, between rich and poor regions); c) Limited vacancies for specialty residency programs compared to candidates numbers; and d) Financial reasons, owing to the very low residency scholarship many physicians instead choose to have several jobs with salaries generally 3–4 times greater than the scholarship [54]–[57].

Our results were also consistent with other studies that focused on the relationship between the lack of proper professional training and the delays in care to STEMI patients. In addition, there was also a consensus among researchers about the need for health curriculum reform in Brazil [58]–[59].

Another issue highlighted by this study was the widespread use of older equipment, specifically electrocardiogram machines. Rapid technological advancement over the past two decades has allowed for almost instant ECG transmission by internet through mobile and wireless technologies. Unfortunately, such technology is not available in most Brazilian hospitals, particularly within primary hospitals where faxing an ECG by phone is the main mode of transmission of an ECG to a PCI center. The quick transmission of an ECG to a PCI center is well known to reduce time from FMC-to-balloon catheter inflation at a PCI center [60]–[64].

Internationally, the recommended door-to-balloon time has decreased to less than 90 minutes within tertiary hospitals [50]. Now the challenge lies in reducing the time encompassing a patient’s presentation to a primary hospital, STEMI diagnosis, and their transfer to the PCI center [52]. Our results were also similar to other studies where it was observed that delays in interhospital transfer of patients was a major predictor of increased ischemic time and higher mortality in the first year [11], [16], [17]. A study performed in Illinois, USA, analyzed the interhospital transfer time of STEMI patients that needed PCI from a non-PCI-capable hospital to PCI-capable hospital and concluded that approximately two thirds of the delays were due to problems related with patient transport and the logistics associated with this transfer [45].

The rapid transfer of patients from primary hospitals to PCI center has proven to be a great challenge for emergency medical services worldwide [16]–[18], [62], [65].

The periodical American Heart Association guidelines publications that have occurred in the past ten years have oriented and improved the health professional practice in the cardiology field [66]. However, the challenge is to overcome the local problems and limitations related with the different factors that may increase the STEMI patient Door-in Door-out delay time in developing countries.

A deeper analysis of these local difficulties is paramount to identify the specific measures to improve the STEMI patients’ treatment mainly in the countries and regions with high rates of STEMI morbidity and mortality. These areas include: ECG transmission capabilities, proper training of physician and nurse team to interpret ECG and identify the STEMI cases, improvement of the contact between patient and the cardiologist, proper inter-hospital transfer of STEMI patients, and continuous program of service quality.

In our work, using SD modeling, the main stages identified as the causes of delays for STEMI patients care within a primary hospital were analyzed and it was demonstrated that an improvement of 40 to 50% in each of these stages would reduce the delay, approximating optimal acceptable levels. It is important to note that the SD model only provides results if all participants, especially the hospital managers, are willing to suggest solutions. In the present work, information was obtained from physicians, nurses and managers from primary hospital allowing a broader viewpoint of the service and its potential problems. Thus, the generated SD model is in agreement with the current operation status of the investigated primary hospital. If new guidelines for treatment to STEMI patients arise or if there are changes in the structure of the service, an updated SD model can be easily generated allowing the identification of the main factors related with the delays and defining the measures to reduce it.

The SD model generated in the present work was created to analyze the delays in treatment of STEMI patients at a primary hospital and it took into account the influence of variables encountered at each stage of the care process. The proposed improvements based on such a model will depend heavily on factors such as the organizational structure and proper training of health professionals.

However, the implementation of the necessary modifications in the primary hospital may be hampered by the limited capacity of the managers of the health services to make decisions under internal and external pressures arising from political requests unrelated to the health system. However, the generated SD model can still be used to orientate these decisions.

As limitation of the present study was not to focus on health professionals other than those that work within the primary hospital, such as professionals in the EMS service or within the PCI center in order to identify other possible factors related with the delay of STEMI patients care at the primary hospital. Future studies could investigate these other services related with STEMI patients care.

The present study indicates that integrating qualitative and quantitative data and SD mediated modeling may contribute to a deeper understanding of the different factors related with delay in STEMI patients’ treatment. We believe this research model can help the decision makers in providing the best care for STEMI patients, to experiment with new prevention policies, look for improvements in the process and consequently decrease the rate of mortality and complications after a cardiac event.

### Ethics Statement

All participants provided a written informed consent to participate in this study. This study was approved by the Institutional Review Board at the State University of Maringa (COPEP/UEM), in Brazil, under the registration number 266/2011.
